# Cholecystectomy in Children: Indications and Timing

**DOI:** 10.3390/children12081052

**Published:** 2025-08-11

**Authors:** Radu Balanescu, Andreea Moga, Laura Balanescu, Mara Untaru, Ruxandra Caragata, Patricia Cimpeanu

**Affiliations:** 1 Department of Pediatric Surgery and Orthopedics, “Carol Davila” University of Medicine and Pharmacy, 050474 Bucharest, Romania; radu.balanescu@umfcd.ro (R.B.); laura.balanescu@umfcd.ro (L.B.); ruxandra.caragata@umfcd.ro (R.C.); 2 Pediatric Surgery Department, “Grigore Alexandrescu” Clinical Emergency Hospital for Children, 011743 Bucharest, Romania; mara.untaru@stud.umfcd.ro (M.U.); ioana-patricia.beznea@drd.umfcd.ro (P.C.)

**Keywords:** cholecystectomy, laparoscopic, pediatric, gallbladder, cholelithiasis

## Abstract

Background: Pediatric cholelithiasis has become increasingly diagnosed, partly due to enhanced imaging accessibility and rising obesity rates. Despite laparoscopic cholecystectomy being the standard treatment, the optimal timing for surgery remains debated, especially in complicated cases. The aim of our study is to analyze the demographic, clinical, and surgical characteristics of pediatric patients undergoing cholecystectomy and to identify the most favorable timing for surgery in terms of outcomes and complications. Material and methods: A retrospective study was conducted on 101 pediatric patients who underwent cholecystectomy between 2015 and 2024 at a tertiary children’s hospital. Patients were categorized based on surgical timing: elective, early (day 1–4), intermediate (day 5–14), and delayed (after day 14). Demographic data, clinical presentation, laboratory values, imaging, operative time, intraoperative findings, and postoperative complications were analyzed. Results: The median age was 15 years, with 64.35% female. Obesity was highly prevalent and significantly associated with choledocholithiasis and pancreatitis. Elective and delayed surgeries (after 14 days) had the shortest operative times (median: 2 h) and the lowest complication rates. Early surgeries (within 4 days) showed longer operative times and a higher incidence of intraoperative difficulties and complications. Histopathological findings did not influence clinical management, suggesting potential for selective examination. Conclusions: Elective or delayed cholecystectomy after a “cool-down” period of 5–14 days provides the most favorable outcomes in pediatric patients with complicated cholelithiasis. Conservative management remains appropriate for asymptomatic cases. A standardized approach to surgical timing may reduce complications and hospital costs

## 1. Introduction

Over the last decade, cholelithiasis has been increasingly diagnosed in pediatric patients, likely due to improved access to imaging modalities, such as ultrasound, computer tomography (CT), or magnetic resonance imaging (MRI) scans [1,2]. This rising trend has been attributed to various risk factors such as obesity, parenteral nutrition, gender, prolonged antibiotic use, gastrointestinal malformations, and hemolytic disorders such as sickle cell anemia, thalassemia, and hereditary spherocytosis [3,4,5]. There is ongoing debate regarding the indications and timing for cholecystectomy in pediatric patients, and the lack of standardized treatment protocols may result in more complex surgeries and increased complications rate. While the procedure is common in adults, cholecystectomy in children requires careful evaluation, as pediatric gallbladder disease often presents differently and may be associated with underlying systemic conditions.

The most frequent indication for cholecystectomy in children is symptomatic acute cholelithiasis, typically presenting with recurrent right upper quadrant or epigastric pain, especially after meals. Performing cholecystectomy early in the course of the disease reduces the risk of complications such as gangrene, perforation, or abscess formation, and minimizes the need for prolonged antibiotic therapy or additional interventions. More severe presentations, such as acute cholecystitis, choledocholithiasis, or gallstone pancreatitis, require surgical intervention to prevent recurrent episodes and complications such as biliary obstruction or infection. Patients are likely to present with fever, leukocytosis, and ultrasound findings such as gallbladder wall thickening or pericholecystic fluid. In such cases, conservative management followed by delayed cholecystectomy is preferred, as attempting surgery during the acute phase may increase the risk of operative complications, including bile duct injury, bleeding, or need for conversion to open surgery. Delaying surgery allows time for the resolution of infection and inflammation, thereby reducing intraoperative complications and facilitating a safer, easier procedure.

Patients with choledocholithiasis, once the stones have been removed endoscopically, are also candidates for cholecystectomy to prevent further episodes, but timing is very important.

In patients with hemolytic disorders even asymptomatic cholelithiasis often warrants elective cholecystectomy due to the significant risk of biliary complications, and prophylactic surgery has been shown to reduce morbidity. Another indication is biliary dyskinesia, a functional gallbladder disorder characterized by chronic abdominal pain and reduced gallbladder ejection fraction on a hepatobiliary iminodiacetic acid (HIDA) scan. In selected cases, cholecystectomy can significantly improve symptoms and quality of life in these patients. Less common indications include gallbladder polyps, congenital anomalies, or suspected neoplasia. Polyps larger than 1 cm or those showing rapid growth are considered at high risk for malignancy and should be excised [6,7]. Early cholecystectomy—defined as surgery performed within 48 to 72 h of symptom onset or during the index hospitalization—has been shown to be both safe and effective in children, with outcomes comparable to, or better than, delayed surgery.

Children with significant comorbidities, poor nutritional status, or coagulopathies may benefit from delayed surgery, allowing for medical optimization prior to general anesthesia and surgery. In cases of diagnostic uncertainty or atypical symptoms, a period of observation may help confirm the diagnosis and avoid unnecessary surgery. Conservative management remains the preferred treatment approach for asymptomatic and uncomplicated cholelithiasis.

Although cholecystectomy is generally considered a straightforward and routine procedure, it can pose significant challenges, with intraoperative and postoperative complications emphasizing the importance of carefully selecting the optimal timing for surgery [8,9]. Important inflammation in Calot’s triangle can lead to dense adhesions between the gallbladder and the surrounding tissues, obscuring anatomical landmarks, complicating the intraoperative dissection [7,10,11]. Postoperative complications are often related to intraoperative findings and as such, some surgeons advocate for a “cool-down” period to allow the inflammation to subside [12].

The aim of this study is to characterize the pediatric population undergoing cholecystectomy and to determine whether an optimal timing for surgery exists. We hypothesize that delayed cholecystectomy is associated with shorter operating time, fewer intraoperative and postoperative complications and decreased hospital length of stay.

## 2. Materials and Methods

We conducted a retrospective cohort study using data collected from January 2015 to December 2024 at the “Grigore Alexandrescu” Emergency Hospital for Children. All patients who were admitted with a diagnosis of cholelithiasis were identified based on the relevant International Classification of Diseases 9th/10th Revision (ICD-9/10) diagnosis codes. Inclusion criteria comprised a confirmed diagnosis of cholelithiasis, admission in the surgical department, and patients undergoing surgery for cholelithiasis. Exclusion criteria included patients diagnosed with cholelithiasis who did not benefit from surgery in our center, incomplete blood test results before surgery, incomplete postoperative follow-up data, and missing intraoperative records. We analyzed the surgical management of these patients and evaluated their clinical outcome

### 2.1. Patients

We collected and analyzed demographic data including age, gender, and weight status (categorized as normal, overweight, or obese). We also documented associated medical conditions such as hematological disorders, diabetes, and history of parenteral nutrition. In addition, we recorded clinical history relevant to gallbladder disease, including prior episodes of biliary colic and complications such as cholecystitis, choledocholithiasis, or pancreatitis.

### 2.2. Laboratory Results

We analyzed blood test results obtained at the time of admission, including liver enzymes (alanine aminotransferase—ALT; aspartate aminotransferase—AST; gamma-glutamyl transferase—GGT), pancreatic enzymes (amylase, lipase), and C-reactive protein (CRP). Abnormal values were noted as elevated and interpreted as indicative of acute hepatocellular injury, pancreatic involvement, or systemic inflammation.

### 2.3. Surgical Management

Treatment options for cholelithiasis were determined based on clinical presentation, comorbidities, and laboratory and imaging findings. Surgical interventions included endoscopic retrograde cholangiopancreatography (ERCP), open or laparoscopic cholecystectomy, and were classified according to timing as urgent, delayed, or elective. Elective surgery was defined as surgery performed in the absence of preoperative complications.

The index admission was defined as the first hospitalization for a cholelithiasis-related complication. Surgeries performed within the first 4 days of the index admission were categorized as early, while delayed surgeries were subdivided into procedures performed on days 5–14 and those performed after day 14.

We also recorded whether surgery was performed after the clinical resolution of inflammation, as indicated by normalized CRP levels and follow-up abdominal ultrasound findings.

The degree of adhesion in Calot’s triangle was noted, especially in patients with a repeated episode of gallbladder inflammation, most commonly due to stones lodged in the gallbladder neck and the cystic duct. Chronic inflammation frequently resulted in dense adhesions, complicating the intraoperative dissection.

### 2.4. Statistical Analysis

Data were compiled and analyzed using Microsoft Excel. Descriptive statistics included the total, counts, medians, and means. Pearson’s correlation coefficients were calculated to assess linear relationships, followed by linear regression modeling using the ordinary least squares (OLS) method. Trends were visualized using scatter plots with regression lines to illustrate associations and patterns in the data.

## 3. Results

Demographic characteristics are summarized in Table 1. A total of 101 pediatric patients underwent cholecystectomy: 36 males (35.64%) and 65 females (64.35%) with a median age of 15 years (range: 10 months to 17 years). Most patients (88.11%) were older than 12 years. Regarding weight distribution, 42.57% were of normal weight, 9.90% were overweight, and 46.53% were classified as obese.

Hematological disorders were identified in six patients (5.94%). Additionally, two patients had a history of parenteral nutrition, and two patients had previously undergone bariatric surgery for weight loss. A positive history of biliary colic was reported in 84.15% of cases.

Patients were categorized into three clinical groups: asymptomatic (n = 28), symptomatic but non-complicated (n = 39), and complicated (n = 32). Complications included cholecystitis (n = 22), choledocholithiasis (n = 19), and pancreatitis (n = 11). There were no cases of gallbladder perforation observed in this cohort.

A significant proportion of patients with complications were classified as obese: 13 out of 22 patients with cholecystitis, 8 out of 19 with cholelithiasis, and 8 out of 11 with pancreatitis. A statistically significant correlation was observed between obesity, choledocholithiasis, pancreatitis, and cholecystitis (*p* = 0.07) (Table 2). All regression models demonstrated a very high degree of correlation, with over 92% of the variance in complication occurrence explained by the presence of obesity. The regression coefficients indicate a strong positive association, and all *p*-values were statistically significant (*p* < 0.001).

Laparoscopic cholecystectomy was the preferred surgical approach in ninety-five cases. Five patients underwent open cholecystectomy, and one patient required conversion to open surgery. Elective cholecystectomy was performed in sixty-three cases. Among patients presenting with complications, eleven underwent ERCP with sphincterotomy prior to definitive surgery. Twenty-four cholecystectomies were performed after resolution of inflammation during the index admission, confirmed by the normalization of CRP levels and improved findings on follow-up abdominal ultrasound.

Laboratory test results showed that on admission, twenty-five patients presented with inflammatory syndrome, forty-six with hepatocellular injury, and fourteen with pancreatic reaction. Preoperatory ultrasound revealed thickening of the gallbladder wall (>5 mm) in nine cases.

When analyzing the group of patients presenting with complications, we found that no patient was operated on in the first 24 h, eight cases were resolved within the first 4 days, fourteen patients underwent surgery between days 5 and 14, and sixteen were operated on after day 16.

The median operative time was 2 h (range: 1–5 h). Elective procedures had the shortest operative time (average: 1.96 h; median: 2 h). Complicated cases, particularly those involving dense adhesions and intraoperative complications, were associated with longer operative times (Table 3).

**Figure 1:**
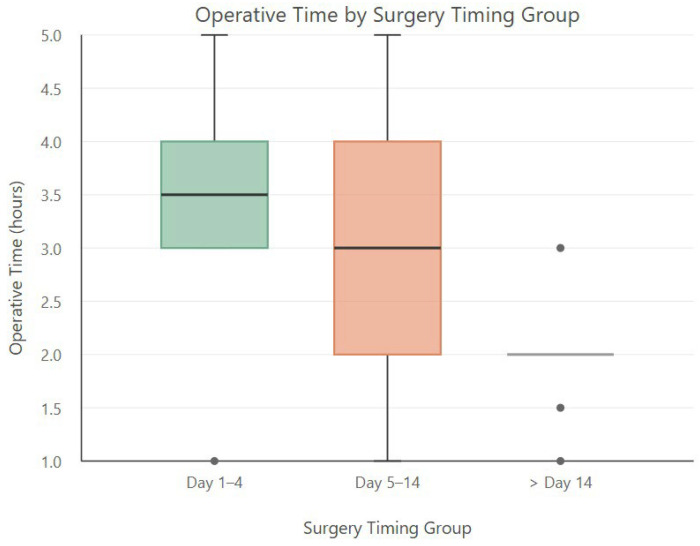
Distribution of operative time (in hours) across different surgery timing groups.

To determine the optimal “cool-down” period following the index admission, we compared median operative times across different surgical timing groups. Notably, patients who underwent surgery after day 14 had a median operative time of 2 h, which was comparable to that of elective cholecystectomies, suggesting that delayed surgery may offer operative advantages once the inflammation has resolved. In contrast, the longest operative times were observed in patients who underwent early cholecystectomy (within days 1–4) (Table 4) (Figure 1).

Operative times by groups were as follows:

- Day 1–4: 3.5 h.
- Day 5–14: 3 h.
- After day: 14–2 h. These findings support the hypothesis that delaying surgery beyond two weeks following the onset of complications allows sufficient time for inflammation to subside, leading to shorter operative times and potentially fewer intraoperative difficulties.

The boxplot analysis demonstrated that surgeries performed after day 14 had shorter and more consistent operative times, while those performed within days 1–4 were associated with longer and more variable durations. An ANOVA test comparing operative times across timing groups yielded F-statistic = 5.81 and *p*-value = 0.0066, indicating a statistically significant difference in operative times between the groups (*p* < 0.01). These results suggest that surgical timing has a significant impact on the length of the procedure.

To identify factors associated with conversion from laparoscopic to open cholecystectomy, a correlation-based analysis was conducted using patient- and surgery-related variables from the full dataset. Pearson’s correlation coefficients were calculated between the conversion and various clinical and perioperative variables, including intraoperative complications, postoperative complications, operative duration, hospitalization time, anatomical factors, and timing of surgery. The strongest correlation was observed between postoperative complications and conversion (r = 0.55), suggesting that complex intraoperative scenarios may contribute both to conversion and to a more difficult postoperative course. Intraoperative complications and anatomical risk factors also showed moderate correlations with conversion. Additionally, early surgery (within the first four days) showed a weaker but consistent trend toward an increased risk of conversion, further supporting the notion that surgical timing plays a role in procedural complexity (Table 5).

Intraoperative findings included Calot’s triangle adhesions in twenty-two cases, gallbladder wall thickening in nineteen cases, and anatomical variations in five patients. Intraoperative complications occurred in five patients, including hemorrhage (n = 2), bile duct injury (n = 2), and gallbladder perforation (n = 1). We conducted an analysis on the impact of intraoperative findings on surgical outcomes and identified several correlations. Adhesions in Calot’s triangle were associated with longer, more complex surgeries, the presence of preoperative cholestatic syndrome, elevated inflammatory markers, early surgical timing within days 1–4, prolonged operative times, and longer hospital stays. These findings suggest that dense adhesions resulting from repeated inflammation significantly contribute to technical difficulty, increased surgical time, and worse short-term outcomes (Table 6).

Postoperative complications were observed in seven patients, including gallbladder bed hematoma (n = 5), hemorrhage (n = 1), and bilioma (n = 1). Of these, three patients underwent surgery within the first four days after admission for complicated cholelithiasis. A statistically significant correlation was identified between the presence of inflammatory syndrome at admission and the occurrence of postoperative complications: correlation coefficient (r): 0.205; *p*-value: 0.040. The correlation is statistically significant at the 0.05 level (*p* < 0.05), indicating a modest but meaningful association between elevated inflammatory markers at admission and a higher risk of postoperative complications. These findings suggest that preoperative inflammatory status may serve as a useful risk stratification factor in surgical planning and patient counseling.

Histopathological examination revealed twelve cases of acute cholecystitis and seventy-eight cases of chronic cholecystitis, while ten patients had no recorded results in our database. No cases of malignancies were identified. Stone analysis was not performed at our center.

## 4. Discussion

Our study provides valuable insights into the management of pediatric cholelithiasis, with a particular focus on identifying the optimal timing for cholecystectomy. As the prevalence of gallstone disease in children and adolescence continues to rise—driven primarily by increasing rates of obesity and improved access to diagnostic imaging—the need for evidence-based surgical timing protocols becomes increasingly important.

Our demographic analysis revealed that most patients were older than 12 years, with a median age of 15, which is slightly higher than values reported in other studies, where a median age of 12 years is more typical. Almost 65% of our patients were female, consistent with the existing literature that shows a higher prevalence of cholelithiasis in females, particularly during adolescents [13].

To further characterize our cohort, we analyzed associated medical conditions. Although rare cases of thalassemia, hereditary spherocytosis or sickle cell anemia and prolonged parenteral nutrition were observed, the majority of patients were either overweight or obese. The increasing prevalence of cholelithiasis in pediatric patients over the past decade has been attributed not only to a greater awareness of gallbladder disease as a source of abdominal pain in this age group but also to the rising rates of pediatric obesity. Although other risk factors such as sepsis, congenital heart disease, prematurity, and intestinal malabsorption have been reported in the literature [9], none were identified in our cohort.

All patients in our study group had their body mass index (BMI) calculated and were categorized as underweight, normal weight, overweight, or obese. Obesity, particularly among females, is a well-established risk factor for gallstone disease. Studies have confirmed that both obesity and female sex are strongly associated with cholelithiasis, with no evidence of false associations [14]. In our series, a significant proportion of complications occurred in obese patients. Our analysis confirmed that obesity is a significant and independent risk factor for the development of cholecystitis, choledocholithiasis, and pancreatitis in pediatric patients undergoing cholecystectomy. The strength of the correlations, as well as the soundness of the regression models suggest that obese children are significantly more likely to develop these complications compared to non-obese patients. These findings support the hypothesis that metabolic and anatomical alterations in obese children may predispose them to more severe disease presentations.

Given the rising prevalence of pediatric obesity globally, this association merits continued attention and highlights the importance of early recognition, risk stratification, and preventive strategies in this population.

Patients with hemolytic diseases require particular consideration. In our study, only one patient with thalassemia experienced a postoperative hemorrhage, but was managed conservatively. While the number of patients with hemolytic disease in our cohort was small, these patients remain at increased risk of gallstone formation due to chronic hemolysis, even in the absence of symptoms.

Gallbladder disease in the pediatric population is becoming increasingly prevalent and can manifest across a wide clinical spectrum, from asymptomatic cases to complicated cases such as cholangitis, choledocholithiasis, and pancreatitis [15,16]. Despite advances in diagnosis imaging and treatment, the optimal timing for cholecystectomy remains controversial, especially when dealing with complicated or recurrent disease.

Laparoscopic cholecystectomy in pediatric patients offers numerous benefits, including reduced postoperative pain, shorter hospital stays, faster recovery, and lower complication rates. As surgical techniques and instrumentation continue to advance, laparoscopy is expected to remain the preferred approach for gallbladder removal in children, reinforcing its role as a safe, efficient, and patient-centered surgical option.

Delaying surgery may increase the risk of complications, morbidity, and healthcare costs. While early surgery may sometimes be necessary in some cases, such as in patients with rapidly deteriorating clinical status, our findings support a delayed approach when feasible, particularly in clinically stable patients. In contrast, elective cholecystectomy can often be performed as a day-case procedure, resulting in minimal cost, reduced patient discomfort, and excellent outcomes. In our study, elective surgery was associated with the shortest operative times, no postoperative complications, and brief hospital stays. Although the indication for surgery in asymptomatic or mildly symptomatic cases remains a topic of discussion, these patients experience the most straightforward surgical course in our cohort.

Controversy typically arises in cases of patients presenting with, for example, pancreatitis or acute cholangitis secondary to gallstone migration. While surgical intervention is clearly indicated in such cases, the optimal timing for cholecystectomy continues to be debated. Although laparoscopic cholecystectomy is now considered the gold standard for treatment [9,10,17,18,19], there is no universal agreement on when surgery should be performed in the context of acute or resolving inflammation. In order to identify the ideal timing for surgery, we divided patients into four groups:

1. Elective surgery that included patients without recent symptoms or complications;
2. Early surgery—within days 1–4 of admission for complicated or symptomatic cholelithiasis;
3. Delayed surgery performed within days 5–14;
4. Delayed surgery—performed after day 14 of admission.

We evaluated several factors contributing to increased surgical complexity and challenges in postoperative management. Statistical analysis showed that the most favorable outcomes were achieved in patients who underwent either elective surgery or, in the case of complicated cholelithiasis, surgery performed during the 5–14-day “cool-down” period, following the index admission for complicated cholelithiasis. This phase involves antibiotic therapy, anti-inflammatory and analgesic management, and the close monitoring of inflammatory markers (particularly CRP), to ensure adequate resolution of the inflammatory process prior to surgery. Although mortality associated with pediatric cholelithiasis is virtually zero, minimizing morbidity, hospital stay, and intraoperative or postoperative risks remains the main goal in surgical planning [20]. Our findings indicate that early surgery (performed within the first four days of admission) is associated with prolonged operative time, challenging intraoperative dissection, and a higher incidence of complications. While postoperative complications were relatively rare, they were more prevalent in patients who underwent early surgery, suggesting that performing surgery during active inflammation may negatively affect both intraoperative safety and postoperative recovery.

Conversion to open surgery was observed primarily in cases involving significant intraoperative challenges, often related to patient-specific anatomical variations, adhesions, or pathological severity. These findings highlight the need for careful preoperative risk stratification, particularly in patients presenting early, with complex ultrasound findings, or with comorbidities such as choledocholithiasis. Recognizing these predictive factors can enhance surgical planning, facilitate more accurate prognosis, and support a more informed consent process, particularly in borderline laparoscopic cases.

Analysis of intraoperative findings revealed that adhesions in Calot’s triangle significantly impact surgery by increasing its complexity and risk. Adhesions are more frequently observed in complicated and inflamed gallbladders, making elective planning less feasible. Adhesions obscure critical anatomical landmarks, requiring meticulous and time-consuming dissection, thus contributing to longer operative times and increased risk of complications. Additionally, patients undergoing difficult dissections often require extended recovery time and closer postoperative monitoring. In severe cases, adhesions may lead to conversion from laparoscopic to open cholecystectomy.

All our patients underwent comprehensive diagnostic workup at admission, including blood tests, abdominal ultrasound, MRI, or CT as needed. For cases involving choledocholithiasis, ERCP with endoscopic sphincterotomy was performed first, followed by laparoscopic cholecystectomy after a “cool-down” period. Although ERCP carries risks such as bleeding, perforation, or sphincter of Oddi dysfunction, no such complications were reported in our cohort [2,11,12,21,22].

Histopathological findings were not significant, with no evidence of tumoral transformation being observed. While this remains a routine procedure, in our study, it did not influence clinical management. These findings are consistent with other reports in the literature, suggesting that routine histopathological evaluation may have limited utility in pediatric cases, without compromising patient care [23]. Although histopathological analysis of gallbladder specimens is routinely performed following cholecystectomy in both adults and children, its clinical value in the pediatric population is increasingly debated. Unlike in adults, where incidental findings such as carcinoma or dysplasia may be detected, the likelihood of discovering clinically relevant, unexpected pathology in children is extremely low [24].

Several retrospective studies in pediatric surgical populations have shown that the vast majority of gallbladder specimens reveal benign and predictable findings, such as chronic cholecystitis, cholesterosis, or mild nonspecific inflammation—diagnoses that rarely influence postoperative management. Moreover, the risk of malignancy in children is exceedingly rare, with only isolated case reports documenting rare neoplasms such as rhabdomyosarcoma or adenocarcinoma arising in gallbladder tissue. These findings raise important questions regarding the cost-effectiveness and necessity of routine histopathological examination in otherwise healthy pediatric patients who present with a clear preoperative diagnosis, normal imaging, and an uncomplicated intraoperative course. In many such cases, histological evaluation merely confirms preoperative clinical and radiographic expectations, offering limited additional diagnostic and therapeutic value [23]. A selective approach to histopathology, guided by clinical risk factors or intraoperative findings, may offer a more resource-conscious strategy without compromising patient care.

Based on our initial findings, a clinical practice guideline is presented in Table 7.

This study has some limitations. Its retrospective design and single-center setting may restrict the generalizability of the findings. Furthermore, the absence of long-term follow-up limits our ability to evaluate recurrence rates or late postoperative complications. Despite these limitations, the consistent trends observed in operative timing and clinical outcomes provide compelling preliminary evidence in support of delayed or elective surgical management in stable patients. These findings highlight the need for future prospective, multicenter studies to confirm our results and potentially establish standardized guidelines for the timing of cholecystectomy in pediatric patients with cholelithiasis.

## 5. Conclusions

Conservative management remains the recommended approach for asymptomatic gallbladder disease in pediatric patients. In contrast, symptomatic cases warrant timely surgical intervention, as delays may increase the risk of complications. For complicated cholelithiasis, initial conservative treatment with antibiotics and anti-inflammatory agents for a minimum of four days is advised to allow for inflammation to subside. Our findings show that delayed surgery, performed after 14 days, results in outcomes comparable to elective procedures, suggesting it may be the optimal strategy in selected cases.

## Figures and Tables

**Table 1:** Demographic characteristics of patients.

Variable	n
Gender	
Male	36 (35.64%)
Female	65 (64.35%)
Age distribution	
Median age	15 years
<3	2
3–5	0
6–8	2
9–11	8
12–14	29
15–17	60
Weight distribution	
Normal weight	43 (42.57%)
Overweight	10 (9.90%)
Obese	47 (46.53%)
Diabetes	0
Hematologic disorders	6 (5.94%)
History of parenteral nutrition	2 (1.98%)
History of weight reduction	2 (1.98%)
History of biliary colic	85 (84.15%)

**Table 2:** Association between obesity and complicated cholelithiasis.

Outcome Variable	R^2^	Coefficient (Obesity)	*p*-Value
Cholecystitis	0.959	0.464	<0.001
Choledocholithiasis	0.960	0.402	<0.001
Pancreatitis	0.923	0.233	<0.001

**Table 3:** Operative time in complicated cases.

	Cholecystitis	Choledocholithiasis	Pancreatitis	Intraoperative Complications	Calot’s Triangle Adhesions	Anatomical Variation	Gallbladder Wall Thickness
Median	3	2	2	3.7	3.63	2	2.93
Average	2.77	2.34	2.63	3.4	3.26	2.4	2.86

**Table 4:** Timing versus time.

	Elective Surgery	Surgery Within Days 1–4	Surgery Within Days 5–14	Surgery After Day 14
Average operative time	1.96	3.37	2.85	2.02
Median	2	3.5	3	2

**Table 5:** Conversion from laparoscopic to open approach.

Variable	Correlation (r)
Postoperative complications (e.g., hematoma, hemorrhage, biliary fistula, bilioma)	0.55
Intraoperative complications (e.g., gallbladder perforation, bile duct injury, bleeding)	0.32
Postoperative hospitalization duration	0.20
Anatomical variations identified intraoperatively	0.19
Overall postoperative complications	0.12
Surgery performed within days 1–4 of admission	0.10
Duration of surgery	0.08

**Table 6:** Correlations of Calot’s triangle adhesions with preoperative and intraoperative findings.

Variable	Correlation (r)	*p*-Value
Duration of surgery (hours)	0.617	8.0 × 10^−12^
Postoperative hospitalization days	0.577	3.3 × 10^−10^
Complication: cholecystitis	0.476	5.7 × 10^−7^
Surgery within days 1–4	0.466	1.0 × 10^−6^
Cholestatic syndrome at admission	0.442	4.2 × 10^−6^
Inflammatory syndrome at admission	0.418	1.5 × 10^−5^
Elective cholecystectomy (negative correlation)	−0.393	5.2 × 10^−5^
Gallbladder wall thickness (intraoperative)	0.353	3.1 × 10^−4^

**Table 7:** Clinical practice guidelines for pediatric cholelithiasis management.

	Clinical Scenario	Recommended Approach	Notes
1.	Diagnosis	- Abdominal ultrasound (first-line treatment) - CT/MRI if inconclusive - Blood tests: LFTs, amylase/lipase, CRP	Confirm presence of gallstones and assess for complications
2.	Asymptomatic cholelithiasis	- Conservative management - Surgery only if hemolytic disease, polyps > 1 cm, or other high-risk findings	Low risk of progression; surgery not routinely indicated
3.	Symptomatic, non-complicated cholelithiasis	- Elective laparoscopic cholecystectomy	Best outcomes: shortest operative time, no complications, minimal hospitalization
4.	Complicated cholelithiasis (e.g., cholecystitis, pancreatitis, choledocholithiasis)	- Initial conservative management (antibiotics, analgesics, CRP monitoring) - ERCP first if choledocholithiasis - Delay cholecystectomy until inflammation resolves	Delaying surgery until after day 14 minimizes intraoperative risks and improves outcomes
5.	Surgical Timing	- Elective: Best outcomes - Early (Day 1–4): Only if clinically unstable - Delayed (Day 5–14): Acceptable compromise - Delayed (>Day 14): Preferred in stable cases	Early surgery increases operative time and complications; delayed or elective preferred when feasible
6.	Preoperative risk stratification	Assess for - Obesity - Elevated CRP - Gallbladder wall thickening - Calot’s triangle adhesions - Hemolytic disease	Predicts surgical complexity; assess surgical planning and obtain adequate consent
7.	Histopathological examination	- Use selective approach based on intraoperative or clinical suspicion	Routine histology has low yield in children; selective strategy improves cost-effectiveness without risk

## Data Availability

The data presented in this study are available on request from the corresponding author. The data are not publicly available due to the privacy.
